# 

**Published:** 1875-05-01

**Authors:** 


### CONTENTS:

*ORIGINAL COMMUNICATIONS:*

Perineal Section. By E. R. Willard, M.D. ---- 237

*TRANSLATIONS:*

Clinical Thermometry. By Dr. B. Kraus. Condensed and Translated by Dr. H. Gradle. 240

*EDITORIAL DEPARTMENT:*

Illinois State Medical Society ---- 245

*CORRESPONDENCE:*

A Letter from Paris. By C. C. Matteson, M.D. 246

*SOCIETY REPORTS:*

Transactions of the Chicago Society of Physicians and Surgeons. Regular Meeting, April 26, 1875 ---- 252

*GLEANINGS FROM OUR EXCHANGES:*

The Cantor Lectures on Alcohol ---- 255

Genesis according to Science (so called) ---- 257

"The Least Sacrifice of Parts," as a Leading Principle of Surgical Practice. By Thomas Bryant, F. R. C. S. ---- 258

On Metro-Peritonitis, following the Use of the Ordinary Female Syringe: A Plea for the Vaginal Irrigator. By Thomas More Madden, M.D., M.R.I.A. ---- 260

*OBITUARY:*

The Late Dr. Addison Niles ---- 265

